# cfDNA Sequencing: Technological Approaches and Bioinformatic Issues

**DOI:** 10.3390/ph14060596

**Published:** 2021-06-21

**Authors:** Elodie Bohers, Pierre-Julien Viailly, Fabrice Jardin

**Affiliations:** INSERM U1245, Henri Becquerel Center, IRIB, Normandy University, 76000 Rouen, France; pierre-julien.viailly@chb.unicancer.fr (P.-J.V.); fabrice.jardin@chb.unicancer.fr (F.J.)

**Keywords:** cell-free DNA, circulating tumoral DNA, sequencing technologies, bioinformatics

## Abstract

In the era of precision medicine, it is crucial to identify molecular alterations that will guide the therapeutic management of patients. In this context, circulating tumoral DNA (ctDNA) released by the tumor in body fluids, like blood, and carrying its molecular characteristics is becoming a powerful biomarker for non-invasive detection and monitoring of cancer. Major recent technological advances, especially in terms of sequencing, have made possible its analysis, the challenge still being its reliable early detection. Different parameters, from the pre-analytical phase to the choice of sequencing technology and bioinformatic tools can influence the sensitivity of ctDNA detection.

## 1. Introduction

Cell free circulating DNA (cfDNA) refers to DNA fragments present outside of cells in body fluids such as plasma, urine, and cerebrospinal fluid (CSF). CfDNA was first identified in 1948 from plasma of healthy individuals [1]. Afterward, studies showed that the quantity of this cfDNA in the blood was increased under pathological conditions such as auto-immune diseases [2] but also cancers [3]. In 1989, Philippe Anker and Maurice Stroun, from the University of Geneva, demonstrated that this cfDNA from cancer patients carries the characteristics of the DNA from tumoral cells [4]. Next, using the recently developed technique of PCR, David Sidransky and his team found the same mutations of *TP53* in bladder tumoral samples and urine pellets from patients [5]. Then, the research and identification of genomic anomalies specific of a cancer type in the circulating DNA, such as *NRAS* and *KRAS* mutations or *HER*-2 amplifications [6,7,8], started to expand, and for the first time, the term of circulating tumor DNA (ctDNA) appeared.

Since the highlighting of this circulating DNA of tumoral origin, technological developments in molecular biology, from quantitative and digital PCR to Next Generation Sequencing, turned it into a powerful liquid biopsy tool. At the era of precision medicine, it seems crucial to identify molecular alterations that will be able to guide the therapeutic management of patients. As tumors release DNA in the blood or other body fluids such as urine, this circulating tumoral DNA, containing the molecular characteristics of the tumor, can be collected with a simple body fluid sample. Since it is minimally invasive, this liquid biopsy is easily repeatable during follow up and in case of relapse. It is also of major interest in some particular cancers where a tumoral biopsy is difficult to obtain such as primary central nervous system lymphoma [9] or cancer subtypes with tissue biopsy containing very little tumoral cells such as Hodgkin lymphoma (HL) for which Reed–Sternbeg cells represent only 0.1 to 2% of the tumoral mass [10,11]. In these particular conditions and malignancies, the sequencing of ctDNA in body fluids could serve as a surrogate for a tumor biopsy. Other body fluids than blood are often used according to the localization of the tumor, such as urine for bladder cancers or cerebrospinal fluid for cerebral tumors [9,12] but blood is the body fluid most often used in studies.

In blood, average cfDNA concentration in healthy individuals can range between 0 and 100 ng/mL of plasma with an average of 30 ng/mL of plasma and is significantly higher in blood of cancer patients, varying between 0 and 1000 ng/mL, with an average of 180 ng/mL [13]. This concentration is correlated with the stage of the cancer, increasing with higher stages, and the size of the tumor. Circulating DNA of tumoral origin represents from 0.01 to more than 90% of the total cell free DNA found in blood [14]. In different types of cancers, a large scale ctDNA sequencing study has shown an association between ctDNA levels and mutational tumor burden [15]. Moreover, given the spatial heterogeneity observed in tumor tissue, ctDNA analysis can determine the complete molecular landscape of a patient’s tumor and give supplementary information on drug targetable alterations and resistant variants [16]. ctDNA kinetics during follow up is correlated with prognosis, as a drastic reduction in its level after treatment is associated with better prognosis, whereas an increase usually means the evolution of drug resistant clones and an ultimate therapeutic failure [17,18,19,20].

Detection of ctDNA during MRD follow up to predict early relapse and at diagnosis in early stages of cancer continues to be a challenge, as the fraction of tumoral DNA contents in total circulating DNA may be <0.01% [21,22]. The development of sequencing technologies being more and more sensitive allows the detection of alterations present in cfDNA at very low variant allele frequencies (VAF), not only for mutational profiling at diagnosis but also for the early detection of disease recurrence and monitoring for therapy response. However, several parameters can affect the sensitivity of ctDNA detection. First, adequate handling of the blood sample, from blood collection to the quality control of the cfDNA extracted, is crucial in analysis. Next, an important step is the choice of the biomarker (s) and the sequencing technology used to detect it. Then, bioinformatic analysis, using error suppression algorithms, is the ultimate tool to discriminate the true variant from false positives.

## 2. Pre-Analytical Requirements

**Figure 1:**
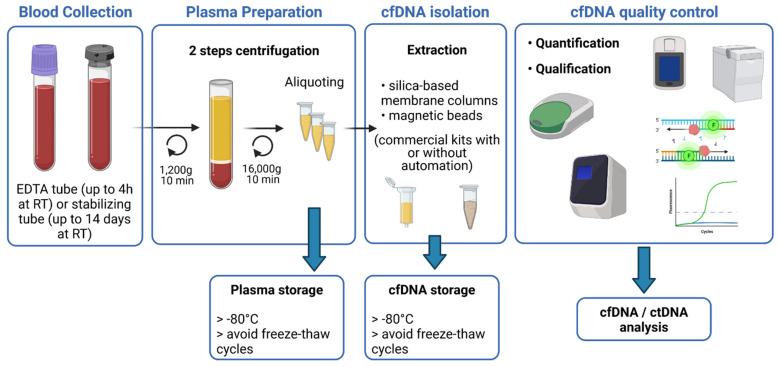
Schematic overview of the main steps for blood sample processing and cfDNA extraction. Blood, collected in EDTA or stabilizing tubes, goes through two rounds of centrifugation to obtain plasma samples. CfDNA is isolated from plasma using commercial kits and is quantified and qualified for further analysis.

Some pre-analytical parameters can affect the sensitivity of ctDNA detection, which strongly relies on input material quantity and quality. Precautions have to be taken to limit degradation of cfDNA and contamination with genomic DNA. Ruptured blood cells were described to be a main source of cfDNA contamination, but it can be in part avoidable by improved pre-analytical processing. The nature of the anticoagulant or stabilizing agent contained in the blood collection tube, the volume of plasma, the preservation, as well as the cfDNA extraction kit, are key elements of the pre-analytical phase, conditioning the accuracy and limit of detection of the analysis [23]. The main steps for blood sample processing are represented in Figure 1.

For optimal extraction of cfDNA from plasma samples, it is recommended to use blood collected into sample collection tubes that provide efficient stabilization of plasma. Several studies have compared different blood collection tubes (BCTs), especially conventional anticoagulant EDTA tubes and as well as long-term storage BCTs from four different manufacturers (such as Streck (cfDNA BCT), Roche Diagnostics (Cell-Free DNA Collection Tube), Qiagen (PAXgene Blood ccfDNA Tube), and Norgen Biotek Corp. (cf-DNA/cf-RNA Preservative tubes)). These last BCTs are pre-coated with preservatives to prevent cell lysis and, therefore, reduce the release of RNA and DNA from hematopoietic cells. All these studies concluded that time between sampling and first centrifugation is a major point when using EDTA collection tubes, and that this time should not exceed 4 h. Whereas, the other BCTs, containing the stabilizing agent, can be stored at room temperature several days without affecting the further analytical performances (up to 14 days recommended but until 3 days for better results). However, despite the presence of stabilizing agents, the ambient temperature must be respected to avoid contamination with normal genomic DNA [24,25,26,27]. Therefore, EDTA tubes are suitable for internal analysis or monocentric studies, but if blood has to be shipped for external analysis, specialized long-term BCTs are more convenient.

Once sampling, blood collection tubes have to be centrifuged for plasma separation. This step can also affect cfDNA concentration and several studies set out to determine the best centrifugation protocol. The two-step centrifugation protocols turned out to be the suitable ones to prevent unwanted release of genomic DNA. Blood cells first have to be removed by slow centrifugation (1200–2000× *g* for 10 min at +4 °C or RT) in order to avoid cell lysis. Whereas afterwards, cellular debris and fragments will be removed by short-term high-speed microcentrifugation of the plasma supernatant (12,000–16,000× *g* for 10 min at +4 °C or RT), either before or after a freeze–thaw cycle [28,29]. The most crucial step is to not disturb the buffy coat while collecting the plasma after the first spin. Plasma samples should then be aliquoted to avoid repeated freeze–thaw cycles and kept at −80 °C for long-term storage.

Yield of cfDNA can also differ according to the extraction kit. Various commercial purification kits have been tested, in particular kits from Qiagen (QIAamp circulating nucleic acid kit and QIAamp min Elute ccfDNA mini kit), Promega (Maxwell RSC ccfDNA plasma kit), Applied Biosystems (Mag MAX cell-free DNA isolation kit), and Norgen Biotek (plasma/serum cell-free circulating DNA Purification midi kit). These kits work either with columns or magnetic beads and some are or can be automated [30,31]. All studies agreed to conclude that Qiagen kits, with or without automation, give the best performances. Once extracted, cfDNA should be stored at −80 °C.

Several studies emphasized the importance to perform consistent quality controls (QC) on the isolated circulating DNA. cfDNA is released through apoptosis and necrosis of normal and malignant cells and is highly fragmented [32]. Its size ranged between 20 and 220 bp with a maximum peak at 167 bp, corresponding to the length of DNA wrapped around a single nucleosome [33]. The use of fluorimetric methods is not suitable to accurately quantify cfDNA as it will not discriminate cfDNA from contaminating genomic DNA (gDNA). Contrariwise, capillary electrophoresis can measure the size of DNA fragments and give an estimation of the absolute concentration of cfDNA [34,35], but will not evaluate the presence of impurities that could inhibit downstream enzymatic reactions. QPCR-based and ddPCR methods can evaluate amplificability of cfDNA, as well as concentration and integrity, but are negatively impacted by gDNA contamination, through distorting the ratio between short and long amplicons [36]. Recently, Alcaide and colleagues developed a promising multiplex ddPCR single-well assay, which can evaluate the quantity, quality, and fragment size distribution of cfDNA samples using low inputs and without the need of reference samples and calibration curves. This assay targets at the same time several olfactory receptor genes, representing three fragment size ranges, and a customizable control diploid locus. Unfortunately, the determination of cfDNA yields can still be affected by gDNA contamination and by copy number alterations [37].

Despite recent promising progresses, the pre-analytical process of blood samples still need standardization and further investigations to improve quality controls of the cfDNA that will be used to detect circulating DNA of tumoral origin.

## 3. Detection of ctDNA by Sequencing Technologies

Sequencing technologies for detection and analysis of the ctDNA range from point mutations analyses using PCR-based methods to analyses of whole genome using NGS based methods. The choice of the method employed depends on the application and the sensitivity intended (see Table 1 for comparison of some selected techniques).

Targeted approaches can detect, with high sensitivity, specificity and at a fast and cost-effective rate, already known recurrent mutations. These hotspot mutations frequently occur in a specific type of tumor and can be, most of the time, targeted by a therapy. Thus, targeted approaches can be very useful for the follow up of minimal residual disease to early detect relapse or track resistant mutations. Contrariwise, untargeted approaches are less sensitive but are useful for the discovery of new DNA mutations and genome wide alterations such as copy number variations (CNV, or copy number alterations, CNA).

Several parameters in the sequencing processing can affect the sensitivity of detection. One of them, also depending in part on the pre analytical process, is to put enough genome equivalent of cfDNA in the sequencing reaction to have enough altered molecule to detect. For example, as around 3000 copies of haploid genome are present in 10 ng of DNA, approximatively 60 ng of cfDNA will be required for a sensitivity of 0.01% (one rare event in 10,000 molecules), which is often challenging, even more if we consider that more than one observation is necessary to determine a true variant. Amplification steps cannot replace low input of cfDNA because the polymerase will introduce errors, increasing the risk to have false positive variants. Another parameter that may improve sensitivity is to monitor multiple alterations simultaneously in order to increase the chances of detecting ctDNA. With a binomial simulation, Van der Pol and Mouliere showed that, in theory and at a given concentration of cfDNA, increasing the number of mutations analyzed could improve the detection of low fraction ctDNA [21]. This kind of analysis was made possible with the advent of next-generation sequencing technologies, by increasing the possibility of multiplexing.

### 3.1. PCR-Based Methods

PCR-based methods, such as the derivatives of qPCR and digital PCR, are fast, cost-effective, and relatively simple to carry out and analyze. They allow detection of single or few mutations at low variants allele frequency, up to 0.1% and less, with high specificity.

#### 3.1.1. Quantitative PCR

At first, the quantitative PCR (qPCR) method, by measuring the fluorescence emitted by a labeled probe during amplification of a targeted gene, was used to estimate the concentration of cfDNA in plasma of patients with cancer [38]. Later, qPCR assays were developed to detect mutations in tumoral cfDNA and the sensitivity of detection was improved by promoting the specific amplification of the mutant allele. Among the most used techniques, we can find ARMS-PCR, PNA-LNA Clamp PCR, or COLD PCR.

ARMS-PCR (amplification-refractory mutation system) is a simple method for detecting point mutations or small deletions, in which DNA is amplified by allele specific primers. In this technique, the lack of 3′ to 5′ exonuclease proofreading activity of the Taq polymerase reduces dramatically the annealing and hence the amplification in case of mismatch at the 3′ end of the primer. The limit of detection for this technique seems very variable according to the studies published, depending on the method, the samples used to determine this threshold or the mutations themselves. Although there are some improvements of the method, the false positive rate is still high with a limit of detection around 0.5 to 1% in plasma samples [39,40]. This limit can go down to 0.015% with ARMS-plus that includes a “Wild-type blocker” and in which amplicons were shortened to 50–80 bp, prohibiting the non-specific amplification and thus increasing the detection specificity [41].

PNA-LNA (peptide nucleic acid-locked nucleic acid) Clamp PCR uses a blocking synthetic nucleic acid analog complementary to wild type sequence to favor the amplification of the mutant allele. This method is particularly used in non-small cell lung cancer (NSCLC) to detect *EGFR* mutations, especially T790M mutations in tumor resistant to EGFR-TKIs (tyrosine kinase inhibitors), where cfDNA could be an alternative to the re-biopsy. This technique shows a high sensitivity with the detection of 0.1% mutant allele and a specificity of 79%. Using smaller PCR products and by increasing the number of cycles, Watanabe and colleagues reached less than 0.1% detection rate [42]. More recently, a dual PNA clamping-mediated LNA-PNA PCR clamp (LNA-dPNA PCR clamp) assay with two PCR rounds of PNA clamping succeeded in achieving a limit of detection of 0.01% [43].

COLD PCR (co-amplification at lower denaturation temperature-PCR) is an amplification method that selectively enriches low-abundance variant alleles from a mixture of wild-type and variation-containing DNA, irrespective of mutation type and position, by exploiting the critical denaturation temperature. The use of a lower denaturation temperature results in selective denaturation of molecules containing wild-type mutant heteroduplexes, which is followed by amplification. COLD-PCR has been used to improve the reliability of a number of different assays that traditionally use conventional PCR, such as Sanger sequencing, pyrosequencing or qPCR, greatly increasing their sensitivity. Thus, this method can detect mutant allele fraction down to 0.1% [44,45].

#### 3.1.2. Digital PCR

As an example in lymphoma, this technique has a potential clinical use in diffuse large B cell lymphoma (DLBCL), as co-occurring mutations in *MYD88* and *CD79B* can predict response to Ibrutinib treatment, thus providing a predictive molecular tool for patient and therapy selection [46]. As well, in primary central nervous system lymphoma (PCNSL), mutation *MYD88* L265P was identified by ddPCR in cerebrospinal fluid or vitreous fluid with a superior sensitivity when compared with qPCR [47,48]. Since this mutation is found in up to 85% of PCNSL cases and not in non-hematological brain tumors, this ddPCR assay may be a promising technique for minimally invasive confirmation of PCNSL diagnosis.

BEAMing (beads, emulsion, amplification, magnetics) is a highly sensitive digital PCR method that combines emulsion PCR and flow cytometry to identify and quantify specific somatic mutations present in DNA [49]. Diehl and coworkers used a BEAMing approach to detect mutations in cfDNA from patients with colorectal cancer, showing that ctDNA dynamics reflects tumor responses and progression, and that ctDNA detection after surgery represented a marker of residual disease [50]. This method, mainly used so far in solid tumors, such as colorectal [51], breast [52], and lung cancers [53], has a highly sensitive detection rate with variant allele fraction as low as 0.01%.

Although ddPCR allows for quantitative assessment of mutant frequencies in cfDNA, it is limited by the number of fluorescent probes that can be used in one assay (up to five) [54,55].

Copy number variations have also been investigating in cfDNA using ddPCR. Even if the number of targets is limited, it can be a useful tool for detecting, simply and rapidly, some gains or losses, which are associated with poor prognosis at diagnosis or during follow-up [56,57].

DdPCR can also be suitable to detect chromosomal rearrangements, especially in hematological malignancies. Among others, assays have been developed for translocation t(11;14) deregulating the *CCND1* gene and translocation t(14;18) deregulating the *BCL2* gene, which are frequently observed in Mantle cell lymphoma (MCL) and follicular lymphoma (FL), respectively [58,59]. The sensitivity of these techniques can go down to 0.01%.

#### 3.1.3. PCR Coupled with Mass Spectrometry

The major limitation of the previous PCR-based approaches is their very limited multiplexing ability. Mass spectrometry-based methods such as surface-enhanced Raman spectroscopy (SERS) and UltraSEEK are adaptation of the conventional PCR method with a unique advantage in multiplexing to detect ctDNA mutations at low frequency with low input amount of cfDNA and fast turnaround time.

SERS is a surface-sensitive technique that enhances Raman scattering by molecules adsorbed on rough metal surfaces or by nanostructures such as plasmonic-magnetic silica nanotubes [60]. The detection of target specific DNA is based on the use of labeled nanotags (Raman reporters) and the measurement of the Shift in the spectrum of Raman reporter that can provide information about low-frequency transitions in molecules. The status of mutations is then analyzed with SERS spectrum where unique spectral peaks demonstrated the presence of targeted mutations. Multiplex PCR/SERS identifying three hotspot mutations has been developed in melanoma and colorectal cancer with a limit of detection as few as 0.1% [61,62].

The UltraSEEK chemistry is able to interrogate multiple informative variants within a single reaction. In this method, the mutant allele is specifically targeted by a primer extension step that omits the wild type allele. Reaction products are subsequently captured to a solid support, washed and released. Eluted products are then submitted to MALDI-TOF Mass Spectrometry. The use of a 68 mutations panel on cfDNA from melanoma patients showed the same sensitivity as ddPCR [63]. In NSCLC, the limit of detection of the UltraSEEK Lung Panel, consists of 73 variants, was 0.125–1% with low input of specific tumoral cfDNA fragments beforehand measured with the LiquidIQ Panel [64]. Of note, this study showed the importance of preanalytical cfDNA quality control and input amount for the accuracy of liquid biopsy testing. The comparison between UltraSEEK and a real-time PCR test (cobas *EGFR* Mutation test v2) showed a concordance of 100% with more than 10 ng of cfDNA, whereas it fell to 73–84% when less than 8 ng were used, implying a loss of sensitivity.

Overall, these PCR-based assays are very effective tools for detecting mutations at a relatively low-cost, which make them feasible in routine clinical practices. The main limitation is the limited multiplexing ability, which restricts the possibility of targets and can lead to a greater consumption of material. Furthermore, the alterations detected must be previously known such as hotspot mutations, which is more suitable for a minimal residual disease but less as a diagnostic tool.

### 3.2. Targeted NGS-Based Methods

Targeted deep sequencing techniques are still limited to a certain number of regions but can cover entire genes or entire coding regions of genes. Thus, they are suitable for genes without hotspot mutations, which is often the case for loss of function mutations in tumor suppressor genes.

Targeted enrichment in library construction can be achieved by direct amplification (amplicon or multiplex PCR) or hybridization capture (hybrid capture) of the DNA regions of interest. Techniques using multiplex PCR-based methods are more dependent on the length of the fragments and may require several simultaneous reactions for target enrichment to cover a large region of a gene, consuming more DNA. Hybrid capture methods employ custom RNA probes complementary to targeted regions and are able to detect both single nucleotide variants (SNV) and structural variants [65]. In this method of enrichment, the fragmentation of cfDNA can lead to a heterogeneous coverage across targeted exons with a lower fragment depth in the edge regions of exons, which must be taken into consideration when designing the panels for ctDNA sequencing [66].

The main issue of going down in sensitivity is the reliability of interpretation in the discrimination between the true and the false variants. Although they have high sensitivity and specificity, NGS platforms show a random error rate between 0.1 and 1.5% per base call, but library preparation protocols have been upgraded to improve the detection of rare variants [67,68]. In targeted DNA sequencing, the use of few DNA molecules combined with ultra-deep sequencing increases the risk to read several times the same molecule where polymerase errors are introduced at any step during the NGS process, leading to the inability to confidently call rare variants. One of the major recent technological advances is the use of molecular barcodes, which are random sequences introduced before any amplification step. They allow the counting of original DNA molecules instead of PCR duplicates, thereby enabling digital sequencing and resulting in unbiased and accurate mutation profiles with an increased sensitivity [69,70,71,72].

- Tagged-amplicon deep sequencing (Tam-Seq)

Tam-Seq is an amplicon method using a target enrichment array with barcoded primers to prepare the amplicon library for NGS. First, an initial targeted preamplification step is carried out, followed by a selective amplification of the regions of interest in single-plex reactions. Then, sequencing adaptors and sample-specific barcodes are attached to the amplicons in a further PCR. It was first able to detect mutations in circulating DNA with high sensitivity and specificity (>97%) at allele frequencies as low as 2% [73]. The technique has been recently improved (enhanced Tam-Seq, eTam-Seq) with a primer design strategy, allowing for amplification of highly fragmented DNA, a workflow reducing the background error rate, and a more efficient calling algorithm with better detection of SNV and indels (insertions/deletions), and also CNV [74]. This assay, using an optimal amount of DNA, detected 94% mutations at 0.25–0.33% allele fraction (AF) with a limit of detection down to 0.02% AF with high per-base specificity (99.9997%). In this study comparison of eTam-Seq with dPCR showed a good concordance between the two techniques, demonstrating the quantitative accuracy of eTAm-Seq technology for reliable detection of mutations at low allele frequency [74].

- Safe-Sequencing System (Safe-SeqS)

This amplicon method was originally described by the group of Bert Vogelstein [69]. It was the first approach using molecular barcodes in DNA sequencing, to increase sensitivity of massively parallel sequencing. In this technique, a unique identifier (UID) is assigned to each template molecule before any amplification. Thereby, PCR fragments with the same UID are considered mutant if more than 95% of them contain the identical mutation. Thus, this method allows a correction of amplification and sequencing errors and can quantify rare mutations with a sensitivity of 0.05% of allele fraction. Safe-SeqS showed high performance in detecting mutations in cfDNA from patients with solid tumors, for molecular profiling as well as real-time monitoring of minimal residual disease [75]. A recent study on three independent cohort of nonmetastatic colorectal cancer, showed a median mutant allele frequency of 0.046% with a minimum of 0.01% [76].

- Duplex sequencing

Duplex sequencing is an improvement of the Safe-SeqS technique [77,78]. In this method, a semi-degenerated double stranded unique barcoded adapter is ligated to a target double stranded DNA. After sequencing, molecules with the duplex adaptors are compared and mutations are retained only if there is a consensus between both strands. Thus, in addition to get rid of PCR and sequencing errors, the advantage of this technique is to identify artifacts due to sample alterations [79] because it can examine both strands individually and the damage to them is usually not identical (error correction by double-stranded consensus sequence). The theoretically sensitivity of this approach to discovering mutants is one molecule among 10^7 which is much higher in accuracy than conventional next-generation sequencing methods [77,78].

Several studies, in various types of cancers, applied this method on plasma cfDNA. In combination with target enrichment using hybrid capture, this approach allowed detection of tumoral fraction at 0.1% and below with high sensitivity and specificity, providing a powerful tool for diagnosis as well as longitudinal monitoring of disease [80,81,82].

- Targeted error correction sequencing (TEC-Seq)

In this technique, molecular barcoding is also used to facilitate the discrimination between true mutations and false positive variants. DNA fragments are tagged each one with a different “exogenous” DNA barcode before any amplification, as for Safe-SeqS, but not only. The start and end genome mapping positions of paired-end sequenced fragments were also used as “endogenous barcodes” to distinguish between individual molecules. This combination of barcodes allows keeping track of each fragment as they are sequenced around 30,000 times [70]. This approach was applied to several type of solid cancers and demonstrated ability for early stage detection. The analytical sensitivity was 100% and 89% for detecting mutations present at 0.2% and 0.1%, respectively, using minimum thresholds of 0.05% in hot-spot positions and 0.1% at all other locations, resulting in a sensitivity of 97.4% overall, and without detection of false positives (less than one error in three million bases sequenced).

- Single primer extension (SPE) with unique molecular barcode

SPE is an amplicon-based method used by QIAGEN in their QIASeq targeted DNA panel kits. This approach uses only one gene specific primer (GSP) for amplification of each genomic region, which makes it less dependent on the size of DNA fragments than PCR using two primers and offers a uniform coverage. As for capture, the first step is a fragmentation step in which the buffer used inhibits fragmentation of the high length fragments of DNA such as contaminating gDNA. The following steps are reparation and ligation of adapters. These adapters will be used for amplification of targeted region (together with GSP) and contain the degenerated molecular barcodes (UMI, Unique Molecular Index). Moreover, given this UMI contains 12 base pairs, it allows a large number of combinations and a very little risk for redundancy [71]. Theorical sensitivity threshold of this technique is 0.5–1% with over 90% sensitivity and a very few number of false positive. Recently, improvement by using duplex UMI adapters lowered the sensitivity up to 0.1–0.2% allele fractions [83].

This technique of deep sequencing, using molecular barcodes to improve accuracy in variant detection, has been used at diagnosis in order to identify actionable genetic alteration with targeted therapies available for treatment or hotspot mutations to be tracked with ddPCR during follow up, with a detection of variant allele frequency down to 1–5% [84,85]. Further investigations are needed to find the real limit of detection of this technology, which may be below 1% as other techniques using molecular barcoding.

This approach also allowed detection of CNV. In PCR-based library construction, amplification introduces biases in further reads count because the amplification factor is dependent on many parameters such as library size, GC content, region length or competition between primers overlapping the same locus. Thus, the use of UMI via the mCNA tool allows the direct count of targeted DNA molecules before any amplification and the detection of CNV in a robust and sensitive way [86].

- Cancer Personalized Profiling by Deep Sequencing (CAPP-Seq)

CAPP-Seq is an ultra-sensitive assay consisting of a hybrid capture-based NGS method developed for ctDNA detection. In this technique, the first important step is to query cancer databases to identify known recurrent mutations for a particular cancer type. Then, biotinylated oligonucleotide probes, named “Selector”, are designed to target large segments of the concerned regions. The protocol is optimized for low DNA levels and sensitivity is increased using deep sequencing [87,88]. The sensitivity is also improved by its ability to detect simultaneously various types of alterations: single nucleotide variants, rearrangements, insertions/deletions, and copy number alterations. It was originally described to detect and monitor lung cancer but was successfully adapted to a broad range of cancers, including different types of solid tumors as well as hematological malignancies such as DLBCL, LF, and HL [10,20,89,90,91].

With this method, ctDNA was detected in blood of NSCLC patients with 96% specificity for mutant allele fraction down to 0.02%. It was improved in 2016, with the use of iDES (Integrated Digital Error Suppression). This iDES-enhanced CAPP-Seq combines CAPP-Seq with duplex barcoding sequencing technology and with a computational algorithm that removes stereotypical errors associated with the CAPP-Seq hybridization step. This improved version of CAPP-Seq has shown a high sensitivity in the detection of EGFR mutations in cfDNA of NSCLC patients, with variant allele frequency as low as 0.004% with >99.99% specificity. Moreover, using duplex sequencing and covering a large number of mutations (≥200), the authors outperformed iDES and managed to detect ctDNA down to 0.00025%, with an input of only 32 ng of cfDNA [92].

- Immunoglobulin high-throughput sequencing (Ig-HTS)

This test was specifically developed for MRD in hematological malignancies. In this method, ultra-deep sequencing of genomic DNA, with a set of locus-specific multiplex PCR covering all possible rearranged IgH, IgK, and IgL receptor gene sequences, firstly identifies the tumor-specific clonotype. Then, this clonotype can be tracked as a specific fingerprint to quantify ctDNA in lymphoma disease monitoring with a sensitivity of approximatively 10-6 [93,94,95]. This technique presents some technical limitations, including the need of tissue biopsy to identify clonotype and difficulties to identify clonotype sequences in some lymphoma types such as DLBCL of the germinal center type and FL because of somatic hypermutation (SHM). Nevertheless, this method has shown high performance in surveillance ctDNA, after complete remission, to identify risk of recurrence before any clinical evidence of disease in most patients (with a median of 3.5 months) [93,94].

This approach was also used for MRD monitoring in DLBCL patients after CAR-T cell therapy, showing correlation with clinical and radiologic outcomes for all the patients tested [96].

### 3.3. Untargeted NGS-Based Methods

As mentioned previously, untargeted approaches, namely whole exome and whole genome sequencing (WES, WGS), are less sensitive than targeted approaches. The sensitivity of these techniques on cfDNA is estimated around 5–10%, as compared to less than 0.1% for a targeted sequencing approach [97], making it difficult to detect rare events, especially in situations of early detection or minimal residual disease. Moreover, these technologies are more expensive and require both very high throughput sequencing equipment and expertise to analyze the large amount of data generated, which makes its implementation in routine practices challenging. However, these approaches may be necessary for the discovery of new alterations in the context of initial profiling at diagnosis, to provide information for the use of more sensitive targeted techniques during disease monitoring. Even if they are not suitable to detect subclonal events, they may be useful, considering intra tumoral heterogeneity, to highlight new drug targets or to track drug resistance clones [98].

WES is, most of the time, limited to coding regions and splicing sites of genes but it is a good compromise for exploration of unknown mutations at a reasonable cost. It can identify both driver and passenger mutations and also can be extended to promoters, untranslated regions, and non-coding DNA of miRNA genes. Even if protein-coding genes constitute only approximately 1.5% of the human genome, they contain a great majority of the disease-causing mutations [99]. The technical feasibility of whole-exome sequencing (WES) on cfDNA has been demonstrated in various solid tumors and some hematological malignancies [98]. Low coverage and sensitivity, compared to targeted NGS technologies does not allow for the detection of rare variants but WES of cfDNA is suitable for mutational analysis of patients with advanced tumors and increased ctDNA fractions (>5% mutant allele fraction). The first exome-wide sequencing analysis of ctDNA was performed to analyze serial plasma samples (before initiating treatment and at disease recurrence), in order to track genomic evolution and response to therapy in patients with metastatic cancer (breast, ovarian, and lung cancer) receiving systemic therapy [100]. These samples contained high percentages of ctDNA (between 5% and 55%) and the average depth of sequencing coverage ranged from 31- to 160-fold. This study showed the possibility to identify candidate genetic alterations driving treatment resistance using cfDNA analysis. These findings largely agreed with additional studies demonstrating that whole-exome sequencing of cfDNA in metastatic patients could serve as a surrogate for tumor genome analysis, considering the difficulties of doing multiple biopsies and the high ctDNA allele frequencies making WES possible [101,102,103,104].

Additionally, given intra tumoral heterogeneity, analysis comparing mutational profile between tumor and cfDNA mostly identified more mutations in cfDNA with a high prevalence of targetable genes. Beyond SNV detection, WES of cfDNA also allowed analysis of mutational signatures, copy number variations, fusion genes, rearrangements, predicted neoantigens, and tumor mutational burden [98].

Contrariwise to WES, WGS technologies is more suitable to detect ctDNA by identifying structural and non-coding variations such as genome-wide copy number aberrations, methylation profiles, and fragmentation patterns.

To override the cost and analysis time limitations caused by WGS, Heitzer and colleagues developed a shallow genome-wide sequencing approach called Plasma-Seq [105]. This method uses an Illumina MiSeq instrument, which is a benchtop high-throughput sequencing platform often available in routine laboratories. This technique does not have a sufficient sequencing resolution to identify SNV but is able to detect CNV in cfDNA at a depth of 0.1×, with a specificity >80% when ctDNA fraction is ≥10%. Recently, this approach of shallow WGS has been successfully used in cfDNA of DLBCL and HL patients to identify copy number patterns that can differentiate the two diseases at diagnosis [106]. These copy number aberrations were also correlated with clinical parameters, and longitudinal analyses showed correlation with disease status. Moreover, the sensitivity and informativity for HL was better in cfDNA than in tumor, as for mutation detection [10,11,106].

Aneuploidy has also been explored with WGS derived techniques such as Fast-SeqS (Fast Aneuploidy Screening Test-Sequencing System) and WALDO (Within Sample Aneuploidy Detection), using a single specific primer pair to amplify dispersed retrotransposon regions throughout the genome (long interspersed nuclear elements (LINEs)) [107,108]. By simulations with synthetic DNA, the bioinformatic tool WALDO showed high performance to detect individual chromosome arm gain or loss with a fraction of ctDNA >5%, and up to 1% of tumoral fraction with a sensitivity of 78%. However, due to their mechanism of detection, these techniques are limited to cancers presenting aneuploidy.

In order to detect genomic rearrangements, Leary et al. developed a technique called PARE (personalized analysis of rearranged ends), which uses WGS mate-paired analysis of the tumoral DNA to identify patient specific genomic rearrangement. This assay is highly sensitive with detection of ctDNA lower than 0.001% of total cfDNA [109]. Analyses, in breast and colorectal cancers, suggest that ctDNA concentrations at levels >0.75% could be detected in the cfDNA of patients with a sensitivity >90% and a specificity >99%, and that even a single copy of rearrangement from ctDNA can be detected without false positives [110]. In a recent study, PARE was employed to detect rearrangements in gastric tumor, which were used to design a quantitative PCR assay targeting rearranged loci for quantitative monitoring in cfDNA. Thus, the authors were able to predict relapse as the presence of postoperative ctDNA was significantly correlated with cancer recurrence within 12 months of surgery [111].

WGS, combined with artificial intelligence, can also identify genome-wide fragmentation patterns in cfDNA. Several studies in different cancer types have shown that these patterns can be used to detect ctDNA in body fluids and with very low plasma ctDNA fraction [112,113]. Indeed, ctDNA fragments are generally shorter and more variable in their length than those found in controls are. Moreover, beyond this difference of size of fragments in cfDNA between healthy individuals and patients with cancer, their location in the genome can be informative of the epigenetic profile of the origin cells. Indeed, the cfDNA fragmentation landscape represents a nucleosome footprint reflecting the cell and tissue of origin, potentially enabling non-invasive diagnosis of cancer type [112]. Recently, Cristiano et al. used this approach for the early detection of ctDNA from 236 patients with various cancers and reported sensitivities ranging from 57 to 99% with a specificity of 98% [114]. This nucleosome footprinting firstly identified by WGS represents nucleosome depletion at transcription start sites of highly expressed genes and the capture of this chromatin accessibility profile was used by CAPP-Seq technology to define gene expression differences and thus determine the cell-of-origin in DLBCL subjects from cfDNA [115].

Among epigenetic alterations, aberrant DNA methylation events can also represent an ideal biomarker for detection and classification of early stage cancer, as they occur early in cancer development, sometimes before the acquisition of SNVs. Multiple liquid biopsy studies have been performed utilizing DNA methylation markers in various cancer types [21]. As whole genome bisulfite sequencing is inefficient due to low recovery and degradation of DNA after bisulfite conversion [116], high cost and limited information recovery given the low genome-wide abundance of CpGs, techniques has been developed to pre-enrich methylated DNA fragments with or without bisulfite treatment. These strategies are either very targeted, as methylation events of interest occur at known, stereotyped positions [117], or larger to identify methylation patterns, which have been shown to enable accurate determination of cell-of-origin from cfDNA and non-invasive cancer classification. For example, a technique for cell-free methylated DNA immunoprecipitation followed by high throughput sequencing (cfMe-DIP) has been developed for genome-wide methylation exploration of bisulfite-free plasma DNA, on low input cfDNA and with enough sensitivity for early detection of cancer [118]. More recently, a semi targeted assay of 595 genomic regions covering 11,787 CpG sites, named PanSeer assay, allowed the detection of five types of cancer in 88% of post-diagnosis patients with a specificity of 96% [119]. Even if the result needs confirmation, the authors also detected cancer in 95% of asymptomatic individuals who were later diagnosed, demonstrating that cancer can be non-invasively detected up to four years before diagnosis. In lymphoma, aberrant promoter methylation patterns detected in cfDNA have been shown to be an independent and significant poor prognostic factor for 5-year overall survival in DLBCL, outperforming existing clinical risk parameters an independent [120,121].

Moreover, as healthy cells also participate to epigenetic changes, it may need to be distinguished from these of cancers cells [21]. Thus, it could be of major interest to combine epigenetic analysis of the entire cfDNA pool with mutational analysis of ctDNA molecules.

## 4. Bioinformatical Methods

While cfDNA seems to be a promising screening tool, it still remains a real challenge for bioinformatics. While common bioinformatics strategies allow variant identification down to 2–5% allele frequency, in most cases, ctDNA accounts for a small fraction of total cfDNA since most of cfDNA is derived from non-cancer cells and especially blood cells. ctDNA fraction can be lower than 0.1%, leading to the detection of somatic mutations at the same level as the sequencing noise. It implies the use of in silico strategies to distinguish true positive variant calls from sequencing noise.

It has been reported from healthy controls that under an allele fraction of 0.02%, more than 50% of sequenced genomic positions had sequencing artifacts [92]. These errors are particularly due to library preparation, the error rates of NGS technologies, and the physical characteristic of the cfDNA fragments.

In addition, there are many tools and therefore many bioinformatics parameters that need to be optimized when analyzing cfDNA samples. While major progress has been made in the harmonization of tumor analyzes with the GATK4 Best Practices Workflows [122], there is not yet an international consensus for bioinformatic cfDNA analysis and research in this area remains very active.

### 4.1. Adapter Contamination

The quality of cfDNA analysis is particularly impacted by adapter contamination. cfDNA fragments could be shorter than usual which may result in the sequencing of adapters due to too many sequencing cycles compared to their lengths. Consequently, these reads could be either unmappable to the reference genome or could have a lower alignment score. These alignment scores are considered by a large number of bioinformatic tools and could finally affect the results of variant caller algorithms. Adapter contamination could be found both in 5′ and in 3′ of sequenced reads.

Many softwares were developed to find and trim adapters, like Cutadapt [123], TagCleaner [124], Trim Galore [125], or Trimmomatic [126]. In general, these algorithms also integrate the trimming of low quality nucleotides and the extraction of molecular barcodes.

### 4.2. Library Biases and Molecular Barcoding

The amplification of the libraries by PCR includes many biases for counting mutated reads because the number of aligned reads is no longer directly proportional to the number of initial unique targeted DNA fragments. The amplification factor of each region is unknown and depends on many parameters such as library size, GC content, or fragment length. This bias is particularly present for samples with low DNA concentration at extraction. Recent advances in library preparation allow the addition of Unique Molecular Identifiers (UMI) to each read. UMI are especially useful to correct library amplification biases by making each DNA molecule in a population of reads distinct.

There are two main bioinformatic approaches to use UMI for cfDNA analysis.

The first one consists in grouping PCR duplicates prior to any downstream analysis by merging sequences harboring the same UMI tag. To perform this task, the most popular tool is UMI-tools [127]. The advantage of this approach is that it allows the use of classic bioinformatic pipelines after deduplicating the reads. It erases amplification biases due to cfDNA characteristics. However, it no longer provides access to essential information such as the amplification factor of each UMI or the discordant mutation calls of reads having the same UMI.

More recent approaches consist in using new bioinformatic algorithms for variant and CNV calling which are able to take into account the information carried by the UMIs after alignment, i.e., at the end of data processing.

For example, the UMI-VarCal algorithm [128] tries to quantify the number of concordant and discordant UMIs for each candidate variant during the variant calling process. Concordant UMIs were defined as number of unique UMIs for which all the reads carrying these UMIs validate the presence of the variant. Conversely, discordant UMIs quantify the number of abnormal substitutions like sequencing or PCR errors. Another example of barcode-aware variant caller is SmCounter [71]. SmCounter uses a barcode level allele probability and UMI counts to reject candidate mutations lacking enough barcodes with good read evidence. These approaches make it possible to distinguish true mutations at low frequency from sequencing noise and is particularly useful for cfDNA analysis.

Many biases due to the amplification step while preparing sequencing libraries prevent the direct quantification of loci copy-number [129]. cfDNA fragments are often shorter than DNA extracted from tissue and make it impossible to use conventional approaches for the detection of CNV such as read-depth algorithms. Recent approaches, like mCNA [86], use the UMI counts instead of read counts to improve high-resolution copy number variation of genes.

### 4.3. Bioinformatics Processing

There is not yet an international consensus for bioinformatic cfDNA analysis pipeline. The bioinformatics tools and parameters must be adapted to the nature of the sequenced samples (quantity of DNA, quality of extraction, integrity of the cfDNA, etc.), to the kits used to prepare libraries, to the presence of UMI in library construction or not, and finally to the sequencing depth. In addition, sequencing biases are often sample specific which requires an objective assessment of sequencing noise at sample level.

However, this variability, specific to each sample, is not incompatible with an objective evaluation of the performance of bioinformatic algorithms. Some first tools, like UMI-Gen [130], allow to create in silico alignment datasets to evaluate the performance of variant calling and filtration tools. UMI-Gen is a UMI-based read simulator, which reproduces targeted sequencing paired-end alignment files (BAM) by estimating sequencing noise from a set of reference BAM files. It is particularly useful for evaluating the performance of variant calling tools because it allows to vary many parameters (sequencing depth, number of initial UMI, etc.) and to insert variants at frequencies of interest during the simulation. It thus makes it possible to optimize bioinformatic pipelines according to the targeted panels or the sequencing technology.

## 5. Conclusions

Many studies have demonstrated that analysis of ctDNA, as a liquid biopsy, is a powerful tool for non-invasive genotyping across various cancer types, in solid tumors, as well as in hematological malignancies. Investigations have shown the possibility to use ctDNA both at diagnosis, for prognosis or targeted therapies, and during longitudinal monitoring of the disease, as a dynamic biomarker of tumor burden during treatment and to detect relapse after treatment. Moreover, liquid biopsy could be a surrogate for tissue biopsy in some particular cases of tumors not accessible for surgery or spread of tumoral mass and metastasis, given the intra tumoral heterogeneity.

Nowadays, the main issue in ctDNA analysis is to go down in sensitivity without generating false positive results, especially for early detection of cancer at diagnosis and relapse. Due to its short fragment length, low quantity, and small fraction in cfDNA [131], reliable detection of ctDNA can still be a major technical challenge. That is why the most suitable ctDNA assay for a specific application has to be chosen according to its analytical performance characteristics [66]. However, recent optimizations in techniques, from standardization of preanalytical processing to the development of high sensitive sequencing technologies with the help of bioinformatical algorithms for error correction, has the potential to detect ctDNA at the molecular level with a great accuracy.

The next step in the near future could be the integration of ctDNA detection assays into prospective multicentric studies and clinical trials to establish its true clinical utility.

## Figures and Tables

**Table 1:** Comparison of some sequencing technologies for ctDNA detection.

Analysis Type		Technique	Sensitivity (LoD)	Targets	Applications	Advantages	Limitations
PCR based methods	qPCR	ARMS-PCR	0.01–0.1%	Hotspot mutation	Cancer detection and monitoring, targetable alterations, some assays approved for clinical use	High specificity and sensitivity, cost effective, rapid, ease of use	No multiplexing, limited to detection of known mutations
		PNA-LNA Clamp PCR					
		COLD PCR					
	digital PCR	ddPCR	0.01–0.1%	Hotspot mutations, gene fusions, CNV	Cancer detection and monitoring, targetable alterations, some assays approved for clinical use	Up to 5 targets, high sensitivity and specificity, absolute quantification, single molecule analysis, cost effective, rapid, ease of use	Limited multiplexing (number of fluorescent colors), limited to detection of known mutations
		BEAMing					
	PCR coupled to spectrometry	SERS	0.1–1%	Known mutations	Cancer detection and monitoring, targetable alterations, for research use	Multiplexing capacity	Limited to detection of known mutations
PCR based methods		UltraSEEK					

NGS based methods	targeted	Tam-Seq	2%	Known and unknown mutations, indels, CNV, chromosomal rearrangements (capture)	Cancer detection and monitoring, classification, targetable alterations, for research use	High specificity	Amplicon methods by multiplex PCR (depend on fragment size), no error correction
		eTam-Seq	0.02%			Error correction	Amplicon methods by multiplex PCR
		Safe-SeqS	0.01–0.05%			Error correction by SSCS	Amplicon methods by multiplex PCR
		Duplex sequencing	0.0001–0.1%			Error correction by DSCS	Amplicon methods by multiplex PCR
		TEC-Seq	0.05–0.1%			Error correction by SSCS, Hybrid capture method (not dependent on fragment size)	Less comprehensive than WGS or WES
		single primer extension (SPE)	0.5–1%			Amplicon methods by SPE (not dependent on fragment size), error correction by SSCS	Less comprehensive than WGS or WES
		SPE-duplex UMI	0.1–0.2%			Error correction by DSCS	Less comprehensive than WGS or WES
		CAPP-Seq	0.02%			Hybrid capture method (not dependent on fragment size)	Need large input, allelic bias (capture), stereotypical errors (hybridization step), less comprehensive than WGS or WES
		iDES eCAPP-Seq	0.00025–0.004%			Error correction by DSCS and correction of stereotypical errors	Less comprehensive than WGS or WES
		Ig-HTS	0.001%	VDJ rearrangements	Non-invasive monitoring, approved for clinical use	Very high sensitivity	Tissue biopsy needed
	Untargeted	WES	5%	Coding regions, intron-exon junctions, promoters, untranslated regions, non-coding DNA of miRNA genes	Cancer detection, monitoring of resistant clones in metastasis, for research use	Mutation discovery and signatures, detection of CNV, fusion genes, rearrangements, predicted neoantigens and Tumor Mutational Burden	Low sensitivity (increasing depth lead to high cost), need bioinformatic expertise
		WGS	5–10%	Structural variants (fragmentation pattern, genome-wide CNV, methylation profile)	Cancer localization and origin, early detection (early and late stage), for research use	Shallow sequencing, genome wide profiling, identification of cancer signatures	Expensive, variable sensitivity (low) and specificity, need bioinformatic expertise, lots of data generated

Abbreviations: PCR—polymerase chain reaction; ARMS—amplification refractory mutation system; qPCR—quantitative real-time PCR; ddPCR—droplet digital PCR; BEAMing—beads, emulsion, amplification, magnetics; SERS—surface-enhanced Raman spectroscopy; PNA/LNA—peptide nucleic acid/locked nucleic acids; NGS—next-generation sequencing; Tam-Seq—Tagged-amplicon deep sequencing; TEC—targeted error correction; CAPP-Seq—Cancer Personalized Profiling by Deep Sequencing; iDES—Integrated Digital Error Suppression; Ig-HTS—Immunoglobulin high-throughput sequencing; WES—whole exome sequencing; WGS—whole genome sequencing; LoD—Limit of Detection; CNV—Copy Number Variation; indels—insertions/deletions; SSCS—single-stranded consensus sequence; DSCS—double-stranded consensus sequence.

## Data Availability

Not applicable.
